# Inhibitory effects and actions of pentacyclic triterpenes upon glycation

**DOI:** 10.7603/s40681-015-0013-x

**Published:** 2015-08-11

**Authors:** Mei-Chin Yin

**Affiliations:** Department of Nutrition, China Medical University, 16th Floor, No. 91, Hsueh-Shih Road, 404 Taichung, Taiwan

**Keywords:** Advanced glycation end-products;, Pentacyclic triterpenes;, Glyoxalase;, Polyol pathway

## Abstract

Pentacyclic triterpenic compounds including asiatic, betulinic, maslinic, oleanolic and ursolic acid occur naturally in many herbs and plant foods. It is well known that these triterpenoids possess anti-oxidative and anti-inflammatory activities. Furthermore, recent *in vitro* and *in vivo* researches indicated that these compounds could inhibit the production of advanced glycation end-products (AGEs). The impact of these triterpenes upon the activity and protein expression of enzymes involved in polyol pathway including aldose reductase and sorbitol dehydrogenase has been examined, and positive results are reported. These studies suggest that certain triterpenes are potent anti-glycative agents, and may benefit the prevention and/or therapy of glycation-related diseases such as diabetes mellitus and Alzheimer’s disease. In this review article, the anti-glycative activity and action mode of certain triterpenes are highlighted. These information may promote the anti-glycative application of these natural compounds.

## 1. Introduction

Glycative stress from excessive production of advanced glycation end-products (AGEs) enhances the pathogenic progression of several chronic diseases such as diabetes, aging and Alzheimer’s disease. Several enzymes such as aldose reductase and glyoxalase I are involved in AGEs formation and accumulation. Thus, any agent(s) with the capability to limit AGE generation, promote AGE degradation, and/or reduce the protein expression and activity of associated enzyme(s) may potentially alleviate glycative stress and delay the development of glycation associated diseases.

## 2. Pentacyclic triterpenes

Triterpenes consist of six isoprene units, and are abundant in the plants in the form of free acids or aglycones [1]. There are more than 80 distinct types of triterpenes have been identified, according to structure and chemical characteristics. Triterpenes are grouped into euphanes, taraxanes, oleananes, lupaneps, ursanes and baccharanes, in which ursanes and oleananes are the major triterpene skeletons in higher plants. In Asian countries including Taiwan, China and Japan, many herbs used for formula or folk medicine are rich in triterpenes and provide medical effects to prevent or treat a variety of diseases [2]. Furthermore, many triterpenes are widely used as edible flavors, pigments, polymers, fibers and glues for food industry or pharmaceuticals. Pentacyclic triterpenes could be further classified into lupane, ursane and oleanane groups. Recently, the bioactivities of certain pentacyclic triterpenoids such as asiatic acid, betulinic acid, corosolic acid, glycyrrhizic acid, maslinic acid, oleanolic acid, ursolic acid, uvaol and erythrodiol have attracted more attention, and been considered as important sources of nutriceuticals and complementary medicines. These pentacyclic triterpenes are present in herbs such as ground ivy (*Glechoma hederacea*), plantain (*Plantago major* L.), thyme (*Thymus vulgaris*), glossy privet (*Ligustrum lucidum* Fructus), and hawthorn fruit (*Crataegi Pinnatifidae* Fructus); fresh fruits such as apple (*Malus domestica* Borkh), carambola (*Averrhoa carambola*), blueberry (*Vaccinum dunalianum*), guava (*Psidium guajava*), calamondin (*Citrus microcarpa Bonge*), persimmon (*Diospyros kaki* L.), and loquat (*Eriobotrya japonica*); and fresh vegetables such as olive (*Olea europaea* L.), gynura (*Gynura bicolor* DC), daylily (*Hemerocallis fulva* L.), basil (*Ocimum basilicum*), water convoevueus (*Ipomoea aquatic*), spinach (*Spinacia oleracea* L.), mahogany (*Toona sinensis*) and leaf mustard (*Brassica juncea*). The content of each pentacyclic triterpene in these edible plant foods is dependent on the species, season, and conditions of cultivation [3-5]. Since the consumption of fresh vegetables and fruits is encouraged for healthy enhancement, more interest has been raised to understand the benefits of these special plant food component(s) upon health and/or disease prevention. Therefore, exploring the bioactivities and action modes of certain pentacyclic triterpenes merits our attention.

## 3. Bioactivities of pentacyclic triterpenes

It has been documented that some pentacyclic triterpenes possess anti-oxidative, anti-inflammatory, anti-cancer and vasodilatory activities [6, 7]. These findings suggest that these pentacyclic triterpenes are potent agents for diseases prevention and/or alleviation.

### 3.1. Anti-oxidative activities

Excessive production of reactive oxygen species (ROS) and reactive nitrogen species (RNS) including superoxide anion, hydrogen peroxide, hydroxyl radical, or nitric oxide has been highly linked to the development and progression of many chronic diseases including aging, diabetes mellitus, cancer, atherosclerosis, Alzheimer’s disease, cirrhosis and Parkinson’s disease [8-10]. It is known that these ROS and RNS, via their free radical property, could directly attack cell apparatus such as mitochondria and impair functions or induce cell death. Furthermore, these ROS and RNS, via acting as signal transduction mediators, could mediate the protein expression of genes encoded for enzymes or factors which are responsible for cell differentiation, growth, migration, invasion and/or apoptosis. These events, in turn, harm the antioxidative defense system, enhance oxidative stress and impair organ functions [11, 12]. In addition, these free radicals could affect both local and systemic immune systems, which consequently promotes inflammatory reactions, and even causes irreversible inflammatory injury [13, 14]. Many cell lines, animals and even human studies highlight the evidence that pentacyclic triterpenes could provide anti-oxidative activities via scavenging free radicals; sparing non-enzymatic antioxidants such as reduced glutathione, ascorbic acid and alpha-tocopherol; increasing the activity of antioxidant enzymes such as superoxide dismutase, catalase, glutathione peroxidase and glutathione S-transferase; as well as suppressing NADPH oxidase, [15, 16]. By executing these above actions, certain pentacyclic triterpenes are able to diminish oxidative stress, which might contribute to delay the development and/or retard the progression of chronic diseases. Meanwhile, more human and even clinical studies are required to further confirm the anti-oxidative protection and application of these triterpenes.

### 3.2. Anti-inflammatory activities

Inflammation is a protective process involving host cells, blood vessels, associated proteins and other mediators which are intended to eliminate the invaders and necrotic cells, as well as to repair tissues. Many T-helper cell type 1 and 2 inflammatory cytokines and chemokines including interleukin (IL)-1, IL-6, c-reactive protein (CRP), tumor necrosis factor (TNF)-alpha, cyclooxygenase (COX)-2, monocyte chemoattractant protein (MCP)-1 and prostaglandin E2 (PGE2), are mediators involved in the host immune and defensive system against stimuli such as pathogens and chemicals [17, 18]. However, under pathological situations such as diabetes mellitus, cardiovascular disease, autoimmune disease and cancer, the overproduction of inflammatory factors causes cytokine imbalance, evokes inflammatory injury, or even leads to tissue destruction. Furthermore, these cytokines and chemokines activate macrophages and/or modulate crucial mediators responsible for pathological processes, which in turn favor the development of acute and chronic diseases. For instance, IL-8 promotes the formation of transforming growth factor (TGF)-beta, which enhances angiogenesis and fibrosis in solid tumors and facilitate cancer metastasis [19, 20]. Thus, the use of appropriate agent(s) with anti-inflammatory effects could decrease the generation of inflammatory stimuli and delay disease progression. The anti-inflammatory effects and possible action modes of several pentacyclic triterpenes in cell lines, animals, and even humans have been reported [21-23]. It is reported that asiatic acid and betulinic acid could regulate nuclear factor-κB (NF-κB) and mitogen-activated protein kinases (MAPK), two important signaling pathways, to lower the production of ROS and inflammatory cytokines [24, 25]. Taraxasterol could reduce TNF-alpha, NO and PGE_2_ levels in sera from mice with endotoxic shock [26]. These studies also indicated that these triterpenes were able to regulate both up-stream and down-stream inflammatory factors including COX-2, and inhibit the protein expression of cytokines and/or chemokines. These evidence support that these pentacyclic triterpenes could alleviate inflammatory stress, and decline the progression of chronic diseases.

### 3.3. Other bio-activities

Anti-tumor. Betulinic acid induces mitochondrial membrane permeabilization and causes apoptosis in prostate cancer cells [27]. Maslinic acid executes its cytotoxic activities toward lung cancer A549 cells through mediating mitochondrial intrinsic apoptotic and hypoxia-inducible factor (HIF)-1alpha pathways [28]. Ursolic and oleanolic acid cause apoptosis in liver cancer cells *via* disturbing mitochondrial membrane ion homeostasis [29]. These findings suggest that these triterpenes are potent anti-cancer agents.

Anti-microorganisms. Asiatic acid and corosolic acid could enhance susceptibility of *P. aeruginosa* biofilms to tobramycin [30]. Ursolic acid, glycyrrhizic acid and their derivatives may protect liver cells against injury induced by hepatitis B or C virus [31, 32]. Ursolic acid and its derivatives could inhibit the growth of several bacteria including *E. coli, S. aureus, P. aeruginosa* and *K. pneumonia* [33]. Apparently, these compounds could be applied for infection prevention or therapy.

Anti-obesity. Ursolic acid may stimulate lipolysis by translocating hormone-sensitive lipase, lowering perilipin A expression, and up-regulating adipose triglyceride lipase in primary culture adipocytes [34]. 18β-Glycyrrhetinic acid inhibits adipogenic differentiation and stimulates lipolysis in matured adipocytes [35].

Based on the above reported bioactivities, these pentacyclic triterpenes are potent medicinal agents and could be considered as candidates for new drug development. So far, more information is also available regarding their activities against glycation, another important pathological factor involved in the progression of many chronic diseases.

## 4. Glycation and chronic diseases

Non-enzymatic glycation with the formation of Maillard reaction products, also known as AGEs, plays an important role in the pathogenesis of many chronic diseases, e.g., diabetes mellitus, Alzheimer’s disease, atherosclerosis, osteoarthritis, inflammatory arthritis and cataracts [36-38]. Any agent(s) with the capability to inhibit the formation of AGEs may potentially diminish glycative stress and delay or retard the progression of glycation-related diseases. It is known that hyperglycemia, a common pathological characteristic of diabetes, promotes glucose metabolism through the polyol pathway [39]. Aldose reductase (AR), the first and rate-limiting enzyme in this pathway, reduces glucose to sorbitol, which could be further converted to fructose by sorbitol dehydrogenase (SDH), the second enzyme in this pathway [40, 41]. This flux through SDH increases fructose formation, and in turn enhances AGE production and promotes microvascular abnormalities [42, 43]. On the other hand, glyoxalase (GLO)-1, part of the glyoxalase system existed in cytosol of cells, metabolizes reactive alpha-carbonyl compounds such as glyoxal and methylglyoxal, and consequently reduces the available precursors of AGEs [44]. Because AR, SDH and GLO-1 are key enzymes involved in endogenous glycative reactions, and responsible for the generation or degradation of AGEs, the development of new drugs to mediate these pathways and mitigate glycative stress should pay more attention to these enzymes. That is, any agent with the ability to suppress the activity and protein expression of AR and SDH, as well as enhance GLO-1 activity and protein expression, may suppress glycative reactions and decrease AGEs production.

## 5. AGEs

AGEs are mainly formed by the reactions between reducing sugars including glucose, ribose and ascorbate, and the amino groups of amino acids from proteins or other moieties from lipids or nucleic acids. Amadori rearrangement is involved in AGEs synthesis and leads to various forms and characteristics. That is, AGEs are formed via a complex cascade of reactions including dehydration, condensation, fragmentation, oxidation, and cyclization. In addition, reactive dicarbonyl compounds such as methylglyoxal are precursors, also endogenous source, for the extracellular generation of AGEs [45], because methylglyoxal could easily react with lysine or arginine residues to produce imidazolone adducts, Nε-(carboxyethyl)lysine (CEL) and other compounds [46]. Thus, AGEs are protein-bound mixtures consist of nitrogen- or oxygen-containing heterocyclic compounds. Besides pentosidine and Nε-(carboxymethyl)lysine (CML), the structures and chemical properties of many AGEs need further studies to be characterized. Although lowering AGEs in circulation and tissues could attenuate systemic glycative stress, both decreasing AGE formation and increasing AGE degradation and excretion are difficult challenges because AGEs possess the properties of irreversibly cross-linked, heterogeneous and insoluble protein aggregates. However, the development of anti-AGEs drug is definitely necessary and important in order to control the high incidence and morbidity of glycation associate diseases. Glycated hemoglobin, CML, glycated albumin, and pentosidine may serve as markers of disease progression [47, 48] because they are common AGEs present in the circulation or organs of patients with diabetes mellitus or Alzheimer’s disease. Circulating AGEs are consisted of two sources: exogenous and endogenous. The former is from the dietary intake; the latter are synthesized in the organs such as kidney or heart under normal and pathological conditions. The intake of foods rich in glycative products enriches the circulating AGE pool and also increases local and systemic glycative stress [49]. Obviously, persons with glycation associated diseases should limit their dietary intake of AGE containing foods in order to avoid the deterioration of existed diseases. Endogenous AGEs may be formed during natural aging for most people. Under normal physical condition, the tissue content of AGEs depends on the kinds and rates of AGE formation and degradation. Usually, AGEs deposit in circulation or organs during natural aging is ascribed to the time-dependent property because advanced glycation is always coupled with oxidative stress. That is, AGEs content under this condition could be decreased as long as oxidative stress has been neutralized [50]. However, the progression of diabetes, renal failure, atherosclerosis and neurodegenerative disease markedly promotes endogenous AGE production. It is reported that hyperglycemia or other pathological conditions such as renal failure accelerate the production and accumulation of AGEs locally or systemically, in which oxidative stress plays an important role to boost AGE generation via glycoxidation and lipid peroxidation [51, 52]. On the other hand, AGE degradation is dependent on its ligation to macrophage scavenger receptors, protein turnover rate and renal capability for clearance [53]. Apparently, glycation is regulated by multiple factors and conditions. The management of glycation associate diseases is complicated and attracts more challenge.

## 6. AGEs in diabetes mellitus

AGEs are effective contributors toward the pathogenesis of diabetes related macro- and microvascular complications, and the circulating AGEs level are positively correlated to the clinical stage of patients with diabetic complications such as nephropathy or cardiomyopathy [54, 55]. Renal tubular and interstitial cells are the most vulnerable targets for increased glycative stress because hyperglycemia stimulates the tubular cells to secrete vasoactive hormones or factors including angiotensin II, transforming growth factor (TGF)-beta and extracellular matrix proteins such as collagen, which in turn facilitate the synthesis of cross-link components and thicken the basement membrane [56]. These events not only increase AGEs formation in the target cells, but also activate intracellular signal transduction pathways including MAPK and NF-κB, and accelerate the generation of ROS, RNS, inflammatory cytokines, fibrotic factor like fibronectin and angiogenic factors like vascular endothelial growth factor [57, 58]. Finally, an organ or a system is malfunctioned and even failure. In addition, circulating AGEs could be reabsorbed and further metabolized by the proximal tubular epithelial cells. Massive AGEs in renal tissue through declining protein breakdown evoke renal cellular hypertrophy, and cause diabetic nephropathy [59]. After stimulated by AGEs, the organs like kidney could further secrete various intracellular second messengers such as nitric oxide synthase, and subsequently induce the expression of adhesion molecules to benefit the progression of fibrosis and angiogenesis [60, 61]. These studies strongly link AGEs to oxidative, inflammatory and fibrotic injury in diabetes. Therefore, decreasing the level of AGEs in circulation and organs is helpful in order to attenuate or delay the occurrence of these complications.

## 7. AGEs in Alzheimer’s disease

The brain of patients with Alzheimer’s disease has two major neuro-pathological hallmarks, extracellular senile plaques and intracellular neurofibrillary tangles. Senile plaques contain beta-amyloid (Aβ) peptide, and neurofibrillary tangles contain hyper-phosphorylated microtubule associated protein tau [62]. AGEs could be found in both neurofibrillary tangles and senile plaques. Furthermore, CML is mainly localized in the cytoplasm of neurons, astrocytes and microglia in the brains of elder people or patients with Alzheimer’s disease [63, 64]. So far, it is still unknown that these AGEs deposited in brain tissue are endogenously synthesized or exogenously from dietary intake, or may be both. However, it is documented that the accumulation of CML and pentosidine, two major glycative products, are highly associated with the progression of Alzheimer’s disease and other aging associated diseases [65, 66]. These literatures strongly suggest that AGEs play crucial roles for the pathogenesis of brain degradation. AGEs are able to induce neuronal cell apoptosis by acting as neurotoxins or inflammatory mediators to stimulate inflammatory cytokines production and enhance inflammatory stress [67, 68]. Thus, AGEs accumulation in brain neurovascular wall not only changes brain functions, but also speeds up the deterioration of dementia including Alzheimer’s disease. The level of CML and its glycation-specific precursor hexitollysine is markedly increased in neurons of patients with Alzheimer’s disease, especially those with intracellular neurofibrillary pathology [69]. It is commonly considered and accepted that oxidative stress destabilizes many macromolecules including sugars, lipids, proteins and DNA, which promotes glycation reactions and causes an early response in many chronic neurodegenerative diseases such as normal aging process and Alzheimer’s disease. Furthermore, the increased hexitollysine or CML in brain could be partially ascribed to lipid peroxidation in this tissue because of the rich lipid and oxygen content [70], which inevitably leads to neuronal dysfunctions. This oxidative-stress hypothesis regarding the pathogenesis of Alzheimer’s disease suggests that AGEs overproduction in brain tissue is accompanied by excessive formation of oxygen-derived free radicals in cerebrovascular disorders [71].

## 8. Receptors for AGEs

The receptor for AGE, also called as RAGE, is expressed in diverse types of cells including endothelial and tubular epithelial cells. RAGEs could engage many ligands associated with distinct pathological processes, which in turn promote the progression of these diseases. AGE is one class of RAGE ligand, and the engagement of AGE with RAGE as occurred in diabetic complications, renal failure or amyloidosis is responsible for glycative stress in these illnesses. So far, enhanced RAGE expression has been reported in various cells under diabetic conditions [72]. It is commonly explained as that hyperglycemia stimulates the production, protein expression, of RAGE ligands, which subsequently interact with AGEs in the circulation and tissues and forms AGERAGE complex. Furthermore, AGEs, from either dietary source or endogenous source, are able to up-regulate RAGE expression [73]. On the other hand, RAGE acts as a signal transduction receptor for Aβ peptide accumulated in the affected brain parenchyma and cerebral vasculature to enhance the development of Alzheimer’s disease. Thus, the increased AGE-RAGE complex activates signaling pathways, aggravates neuro-inflammation, and finally impairs memory and learning [74]. Meanwhile, RAGE’s ligands are also consisted of S100/calgranulins, high-mobility group box 1 and beta-sheet fibrils, and the reactions between RGAE and these ligands generate pro-inflammatory and prothrombotic molecules and ROS. These events eventually augment oxidative and inflammatory injury, and thrombotic risk in the target tissues, such as atherosclerotic plaques and cardiac infarction [75, 76]. Besides endothelial and epithelial cells, RAGE and its ligands are also presented in tumor cells, neurons cells, podocytes, and smooth muscle cells. The engagement of RAGE and its ligands may trigger diverse signaling cascades including p21ras, ERK1/2, p38 JNK, and Jak/STAT in these targets [77, 78]. Although RAGEs and their ligands play independent roles in the pathology of several illnesses, AGE-RAGE interaction still attracts more attention because it directly activates many crucial signaling mechanisms. It has been indicated that AGE-RAGE interaction stimulates O2- production and raises oxidative stress [79], induces vascular inflammation and thrombosis via activating NF-κB [80, 81], and up-regulates the protein expression of adhesion molecules, chemokines, pro-inflammatory cytokines, matrix metalloproteinases and RAGE itself [82, 83]. Consequently, NF-κB elicits the expression of downstream genes encoded for TNF-alpha, IL-6 and MCP-1, which promote inflammatory reactions and cause irreversible impairment in the target tissues [84, 85]. Glycative stress from AGEs, RAGEs and their interaction is a contributor toward the progression of diabetes mellitus and Alzheimer’s disease; however, the impact of this AGE-RAGE axis upon other diseases like cancer could not be ignored. For instance, ROS and cytokines generated from this axis lead to DNA oxidative and inflammatory damage, and may initialize carcinogenesis. Besides AGEs and Aβ, RAGE can bind to other ligands such as low-density lipoprotein and calgranulins. Apparently, the impact of AGEs, RAGEs, RAGE ligands and their interactions upon human health is not limited to glycative stress. Therefore, any strategy against glycation-associated chronic diseases must consider: (1) lowering the exogenous and endogenous levels of AGEs in the circulation; (2) decreasing the available other ligands of RAGE in the circulation; and (3) interfering the interaction of RAGE and its ligands. That is, any possible inhibitor(s) with the capability to block AGE formation, decline RAGE expression, or interrupt the AGE-RAGE interaction could be considered as a potent candidate for treating diabetes mellitus, Alzheimer’s disease, and other glycation-related diseases.

## 9. Anti-glycative potential of pentacyclic triterpenes

Circulating AGEs could be from the dietary intake and from endogenous generation. Many foods are rich in pentosidine and/or furosine, and some of them are processed by sugar, heat or certain sauces [86, 87]. It is reported that foods cooked by baking or deep frying also contain high AGE levels, in which high temperature, lipids and proteins participate Maillard reactions and lead to the synthesis of various forms of AGEs [88, 89]. Apparently, in order to decrease circulating levels of AGEs from exogenous sources, the consumption of foods rich in glycative products should be limited, especially for patients with diabetes or Alzheimer’s disease. On the other hand, endogenous AGEs could be formed between reducing sugars and amino acids present in the circulation and tissues. The pool of reducing sugars and amino acids in the human body is large and unlimited, unfortunately. Thus, lowering the intake of reducing sugars or amino acids may not make any sense for improving health because this limitation definitely impairs nutritional status. The other alternative is to ingest other natural compound(s) with anti-glycative effects to counteract endogenous AGE formation and/or mediate AGE metabolism. Recently, *in vitro* inhibitory effects of some pentacyclic triterpenes such as astragalosides, boswellic acid and corosolic acid upon the formation of AGEs or their precursors like methylglyoxal and CML have been reported [90-92]. The results suggest that these pentacyclic triterpenes may halt the interactions between reducing sugars and amino acids, decreasing AGE generation and attenuating glycative stress via non-enzymatic actions. Both oxidative and inflammatory reactions benefit glycative processes, and the anti-oxidative and anti-inflammatory activities of several pentacyclic triterpenes such as ursolic acid, oleanolic acid, corosolic acid, maslinic acid, glycyrrhizic acid and erythrodiol have been already demonstrated in rodents [93-96]. Thus, it is highly possible that these pentacyclic triterpenes directly decrease oxidative and inflammatory stress, which in turn and indirectly mitigates glycative stress. These findings, at least, agreed that these compounds were potent agents and could be considered for anti-glycative protection. The other possibility is to explore the agent(s) with the ability to mediate AR, SDH or GLI, which subsequently reduces the production of AGEs or increases the degradation of AGEs. It is reported that oleanolic and ursolic acid could inhibit the activity and/or protein expression of AR and SDH, two major enzymes in polyol pathway, which consequently lowered AGEs levels in kidney, liver or brain [97-99]. Furthermore, AGEs metabolism could be facilitated by up-regulating GLO-1. Other studies revealed that protocatechuic acid and glycyrrhizic acid could enhance the expression of GLO-1, and lowered the level of fructose, methylglyoxal and CML in brain of aging mice or kidney of diabetic mice [99, 100]. These studies suggest that endogenous AGE generation could be suppressed by certain pentacyclic triterpenes, which finally contributes to diminish glycative stress under those pathological conditions. The third possibility is to discover the anti-RAGE agent(s). It is strongly convincible that suppressing RAGE expression and/or interrupting the AGERAGE interaction could more efficiently block glycative reactions and retard pathological progression in glycation associated diseases. A cell line study indicated that glycyrrhizic acid was able to down-regulate RAGE expression [101]. Ursolic acid could decline RAGE expression in brain of aging mice [102]. The decreased RAGE expression could definitely lower AGERAGE complex and diminish glycative stress. Although the support from in vivo studies regarding the anti-RAGE effects of pentacyclic triterpenes might not be sufficient, the effort toward this direction should be encouraged because the anti-RAGE agent(s) may provide multiple medical benefits. In addition, human studies and clinical trial for these pentacyclic triterpenes regarding their anti-glycative effects and action modes will be highly beneficial to demonstrate their effects and elucidate the actions. Certainly, the exploration of other non-triterpenoic antiglycative agents is also warranted to fight glycation-associated disease.

## 10. Blood-brain barrier

One crucial challenge of researches regarding pentacyclic triterpenes is their bioavailability. It is a common and important sense that the development of any pharmacological agent against Alzheimer’s disease has to consider whether this substance could pass through the blood-brain barrier, tightly packed layers of endothelial cells, which surrounds the brain to block high-molecular- weight molecules from penetrating it. This blood-brain barrier mainly acts to block the influx of intravascular substances from the circulation to the brain, and also mediates the transport of substances from brain to circulation *via* several transport systems such as carrier-mediated transport, active efflux transport and receptor-mediated transport [103, 104]. Furthermore, blood-brain barrier is essential for maintaining brain Aβ homeostasis and regulating Aβ transport [105]. Obviously, it is definitely essential to examine whether pentacyclic triterpenes with the capability to pass through the blood-brain barrier.

## 11. Conclusion

Pentacyclic triterpenes are compounds naturally occurring in many plant foods. Based on their anti-oxidative and anti-inflammatory activities, regulation upon AR, SDH and GLO-1, these agents may improve glycative stress. Future studies should probe the effects and action modes of these pentacyclic triterpenes upon RAGEs, AGE-RAGE interaction and blood-brain barrier penetration. These information could enhance the application of these agents for prevention and attenuation of glycation-associated diseases including, but not limiting, diabetes mellitus and Alzheimer’s disease.
